# Fifty shades of green and blue: autopsy findings after administration of xenobiotics

**DOI:** 10.1007/s12024-024-00903-7

**Published:** 2024-10-30

**Authors:** J. Baumgarten, I. Greb, F. Holz, C. Nieß, S. Petzel-Witt, Christoph G. Birngruber

**Affiliations:** 1 Institute of Legal Medicine, University Hospital Frankfurt, Goethe-University, Kennedyallee 104, 60596 Frankfurt am Main, Germany; 2 Poison Control Center, Charité - Universitätsmedizin Berlin, Berlin, Germany

**Keywords:** Methylene blue, Toluidine blue, Organ color, Turquoise, Shock treatment

## Abstract

Unusual findings during an autopsy may come from peculiarities in the position, shape, size, weight, consistency, smell or color of organs. The following study was triggered by an autopsy case in which an unusual blue-green discoloration of organs, which changed during the autopsy, was noticed. A review of the local autopsy database, selected cases including the antemortem clinical documentation and the literature has been performed to clarify the etiology of these conspicuous discolorations and to evaluate their diagnostic value. The study showed that certain xenobiotics may lead to such discoloration. After systemic administration of methylene blue, darkening blue-green discoloration of organs, especially the brain and heart, can be observed. In addition, the systemic administration of toluidine blue also appears to be capable of causing such discoloration. Beyond that, drugs (like Rohypnol^®^) or other foreign substances (like detergents) containing warning colors, i.e. indigocarmin (E132) or Brilliant Blue FCF (E133) may cause discolorations of the upper gastrointestinal tract or the urinary bladder respectively. A blue-green, possibly darkening discoloration of organs during autopsy may point towards an antemortem administration of certain xenobiotics. The affected organs give an indication of the possible route of application and the type of substance. A differentiated interpretation of the etiology of such conspicuous discolorations at autopsy should only be made considering the (medical) history and, if necessary, complementary (toxicological) examinations.

## Introduction

The quotation attributed to Johann Wolfgang von Goethe, “What is the hardest thing of all? What seems the easiest to you: To see with your eyes what is before your eyes” is valid for all aspects of diagnostic medicine and is particularly important for the macroscopic assessment of findings during autopsy. In the setting of a postmortem examination, it is essential to perceive an existing final condition, to describe it objectively, to separate the relevant from the irrelevant, to recognize and name findings and to draw scientifically sound and comprehensible causal conclusions. If unusual findings occur that are not immediately obvious to the examiner, they should be further investigated: To be able to deal with the given case thoroughly, and to be prepared for future examinations.

Based on the principles of patho-anatomical assessment, such unusual findings can for example arise from peculiarities in the position, shape, size, weight, consistency, smell or color of organs [1]. The following study was triggered by an autopsy case in which an unusual green-blue coloration of individual organs or their surfaces, which changed during the autopsy, was noticed.

## Background: the index case (case 1)

**Fig. 1:**
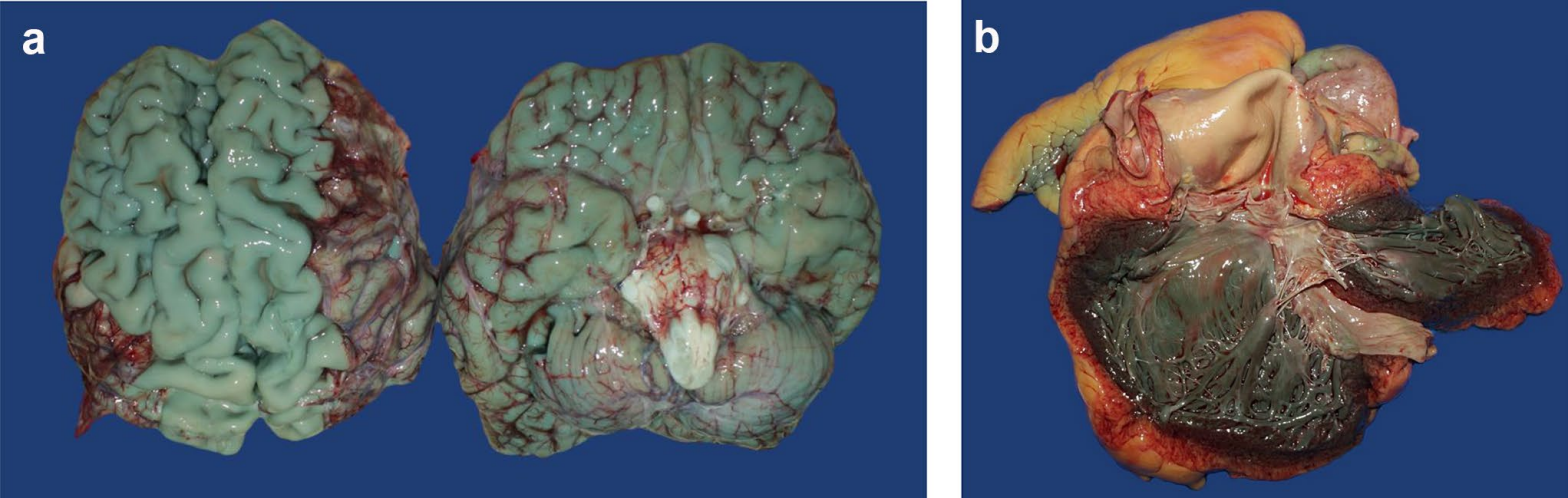
(**a**) The surface of the brain, several minutes after exenteration (case 1). (**b**) The inner lining of the heart, several minutes after opening (case 1)

During the autopsy of the corpse of a 72-year-old man, a subtle bluish coloration of the surfaces of some organs was visible after opening the body cavities. The colorations began to darken after a few seconds and turned into a strong green-blue discoloration. This also affected the brain and heart on dissection (Fig. 1a and b) as well as the mucosa of the esophagus, stomach and duodenum. The man had been admitted to hospital due to decompensated heart failure. During the diagnostic assessment, a perforated duodenal ulcer was seen and treated endoscopically. This was followed by peritonitis with ICU treatment and septic multi-organ failure. The autopsy revealed purulent peritonitis in conjunction with a morbidly compromised heart as the cause of death. The reason for the unusual coloration remained unclear.

## Materials and methods

On the quest for possible explanations for this coloration, a search was first carried out in the institute’s autopsy register Forensik^®^. A total of 15,820 autopsy files between 01.01.2000 and 31.12.2023 were searched in the “main findings” box for the terms “blue-green”, “green-blue”, “bluish”, “greenish” or “turquoise”. After excluding cases in which the match was not based on the description of organ colors or body fluids, eleven cases remained, including the index case.

First, the corresponding protocols and records were evaluated. As no detailed medical treatment records were available for any of the cases in which death had occurred in hospital, in a next step, the medical files have been requested. After evaluation of the cases including the medical files, a literature search was conducted via pubmed. The search terms used were based on the information that has been gained during the review of the cases.

## Results

### Review of the autopsy cases

#### Case 2

**Fig. 2:**
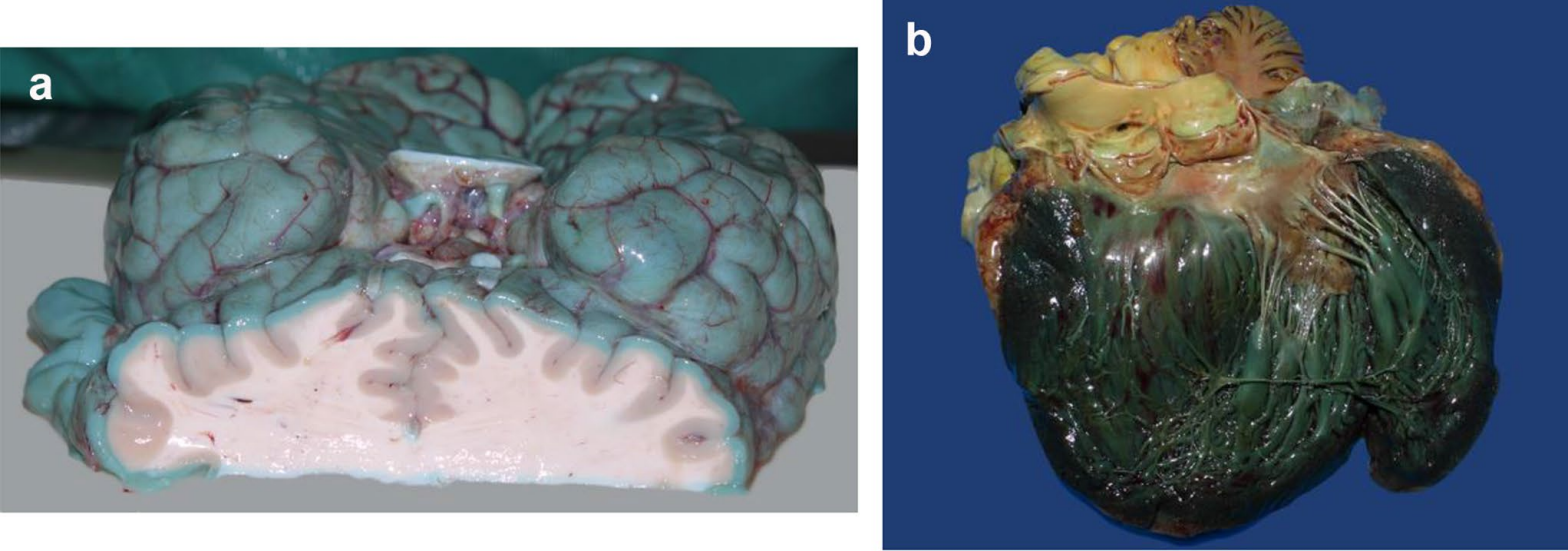
(**a**) The difference between the surface and the fresh cutting surface (case 2). (**b**) Darkened, intense blue-green inner lining of the heart (case 2)

A 30-year-old man received ICU treatment in hospital due to a severe SARS-CoV-2 infection. During the stay he developed acute necrotizing pancreatitis and died shortly after. At necropsy, a slight bluish discoloration of the brain surface and the surfaces of the heart and lungs was noticed when opening the body cavities. Within a short time, a greenish-blue darkening occurred, which happened to the surfaces of the brain (Fig. 2a) and the inner layer of the heart (Fig. 2b) but was not observed in the lungs. Cause of death was an extensive peritonitis following necrotizing pancreatitis.

#### Case 3

A 71-year-old patient had extensive surgery due to bladder carcinoma. During the operation, the spleen was injured, and hemorrhagic shock occurred, which was treated medically. On the fifth postoperative day, the patient died in the intensive care unit (ICU) after frustrated resuscitation with pulseless electrical activity. Clinically, a Tako-Tsubo cardiomyopathy was suspected as the cause of death. At necropsy, a subtle and rapidly increasing green-blue coloration of the surfaces of the brain, heart and pancreas was noted. The darkening green-blue discoloration also affected the cut surfaces of the brain and heart, but not those of the pancreas. Further findings indicated a fatal reinfarction of the heart.

#### Case 4

A 79-year-old woman with dementia drank an unknown quantity of a blue colored cleaning agent containing formalin, became restless, cold-sweaty and vomited. Mucosal damage in the esophagus, stomach and duodenum were diagnosed in hospital. Death occurred the following day on ICU due to therapy refractory lactic acidosis. The autopsy revealed mucosal necrosis in the upper gastrointestinal tract, hemorrhages in the tracheal mucosa and cloudy changes in the lungs as consequences of the detergent ingestion and aspiration. A green-blue discoloration of the urinary bladder mucosa was noted. Ingestion and aspiration of the formalin-containing cleaning agent with consecutive mucosal necrosis, pneumonitis and acidosis was the cause of death.

#### Case 5

A 73-year-old woman had a colon perforation during a colonoscopy, which was sutured immediately. Penicillin was given to prevent infection, which led to anaphylactic shock. Attempts at intubation were unsuccessful, and when a tracheotomy was performed, there was unstoppable bleeding with circulatory arrest. An anaphylactic shock causing death was stated in the death certificate. The autopsy revealed a swollen pharyngeal mucosa, a tracheal incision and blood in the trachea and bronchi with foci of hematemesis in the lungs. In the sigmoid colon, there was a hemorrhagic mucosal injury with surrounding green-blue discoloration of the mucosa. A combination of anaphylactic shock, blood loss and inhalation after tracheotomy was assumed as cause of death.

#### Case 6

A 40-year-old man was hospitalized due to a SARS-CoV-2 infection. A perforation of the heart during the implantation of an ECMO was immediately treated surgically. Postoperatively, there was a high need for catecholamines. The patient then died suddenly in the ICU. The autopsy revealed a greenish discoloration of the surface of the brain and its cut surfaces. The cause of death was multi-organ failure due to a SARS-CoV-2 infection.

#### Case 7

**Fig. 3:**
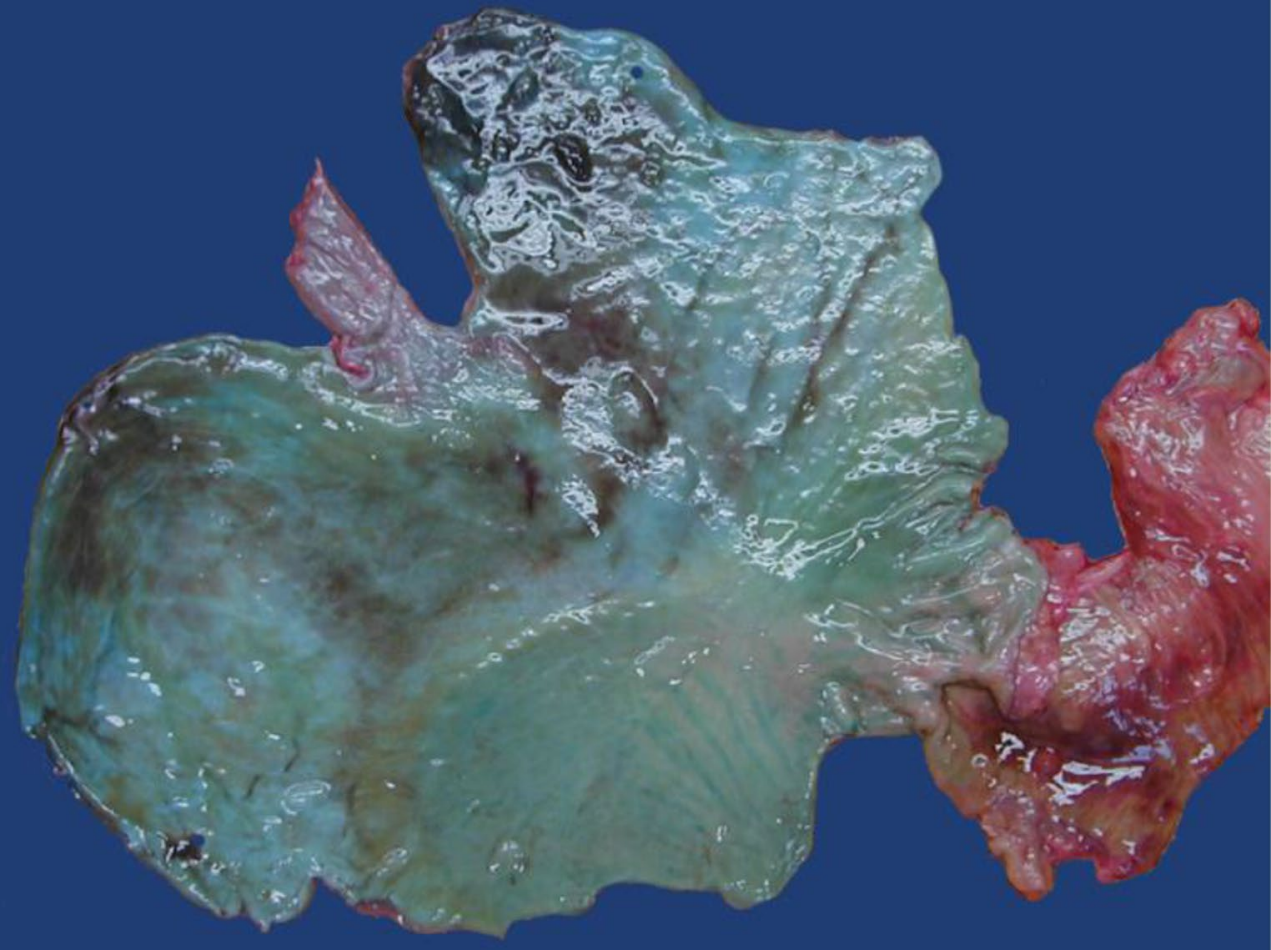
Blue-green color of the gastric mucosa (case 7)

A 46-year-old woman was found lying dead in bluish-colored vomit in her apartment. Empty bottles of alcohol and an empty packet of the drug Rohypnol^®^ (flunitrazepam) had been found in the apartment. An unspecified intoxication was assumed as the cause of death in the death certificate. During the autopsy, 350 ml of green-blue liquid was found in the stomach whose wall was discolored green-blue (Fig. 3), which was considered the result of Rohypnol^®^ ingestion. Forensic toxicological examinations revealed mixed intoxication with alcohol and flunitrazepam as the cause of death.

#### Case 8

A 30-year-old woman had been found dead in her apartment with a plastic bag pulled over her head and taped to her neck. Next to her were empty packs of antidepressants, neuroleptics, and Rohypnol^®^. Initially suffocation was assumed to be the cause of death. The autopsy revealed a less conspicuous green-blue fluid in the stomach and duodenum, accompanied by a green-blue discoloration of the gastric wall and the duodenal mucosa. Forensic-toxicological examinations revealed a mixed intoxication with flunitrazepam, mirtazapine and olanzapine which was assumed to be the cause of death in combination with asphyxia.

#### Case 9

A 71-year-old patient was found lifeless in her bed on the second postoperative day after a total knee arthroplasty. After initially successful resuscitation, she received ICU treatment but died on the same day. No information on the presumed cause of death was given in the death certificate. The autopsy revealed acute right heart failure due to pre-existing pathological alterations to the heart and lungs as the cause of death. A green-blue to turquoise coloration of the cut surfaces of the brain and a turquoise discoloration of the transition zone between the heart muscles and the subepicardial fatty tissue were noticed.

#### Case 10

**Fig. 4:**
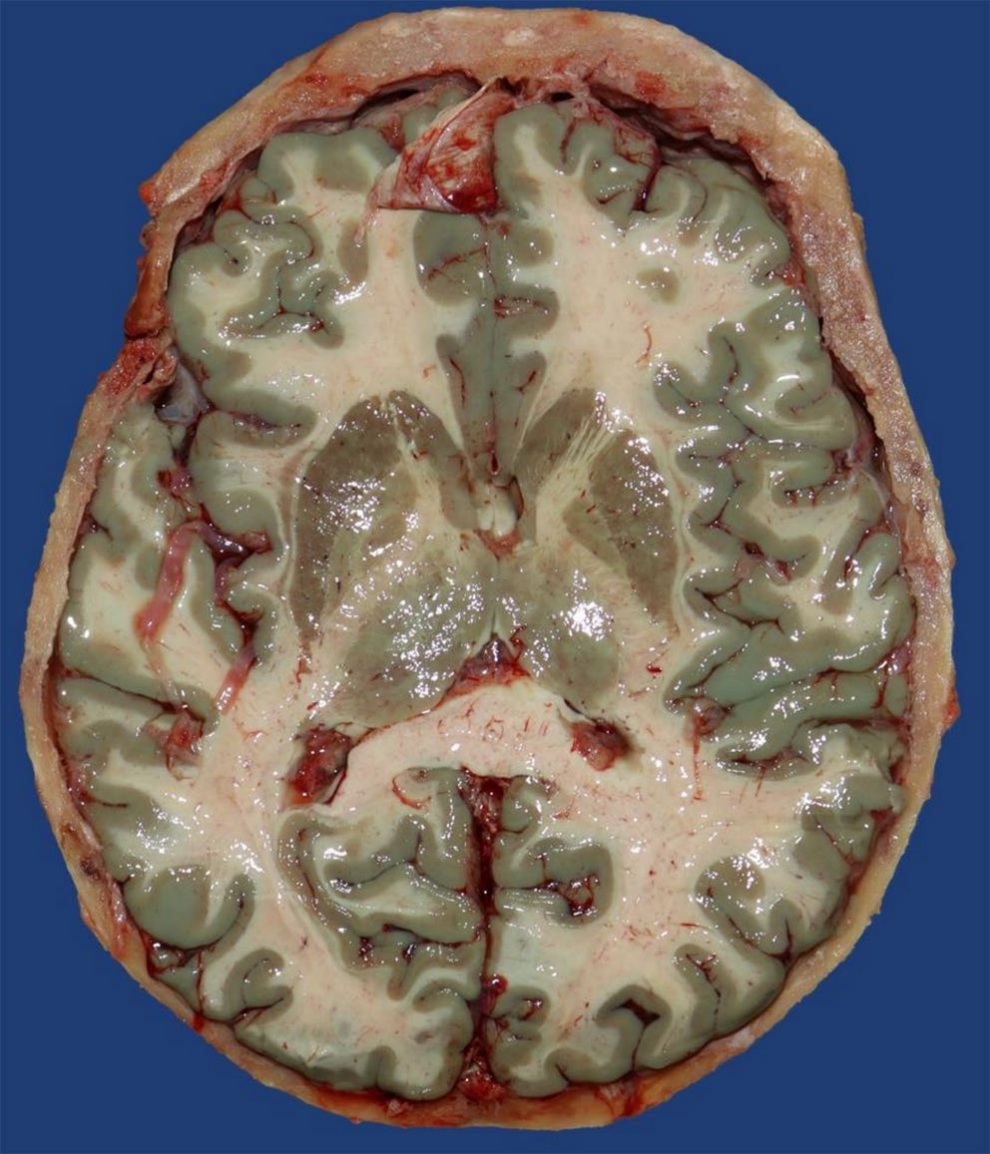
Slight blue-green coloration of cortex and thalamus, moments after the Flechsig cut (case 10)

A 79-year-old man underwent surgery for painful spinal stenosis. On the fifth postoperative day, he developed a fever and his general condition deteriorated despite ICU treatment. In accordance with the patient’s presumed will, the treatment was limited, and he died of sepsis. The autopsy revealed a Waterhouse-Friedrichsen syndrome as cause of death. An initially subtle green-blue discoloration (Fig. 4), which darkened to a strong green-blue color, of the surface and the cut surfaces of the brain and the heart as well as the endocard was noticed.

#### Case 11

A 75-year-old man was hospitalized after out-of-hospital cardiopulmonary resuscitation. Because of the acute myocardial infarction, a cardiac catheterization procedure with stenting took place. Post intervention, high catecholamine requirement persisted, and the man died the following day. Multi-organ failure after myocardial infarction with prolonged shock was seen as the cause of death. The autopsy revealed an extensive posterior wall infarction as the cause of death. An incidental finding was blue urine in the pouch of a urinary bladder catheter that was still in place.

### Review of the medical files

To complement the review of the autopsy cases, the medical files have been requested for the 9 cases, in which death had occurred in the hospital. In 6 of these 9 cases (case 2, 3, 6, 9, 10, 11), the drug methylene blue (MB) had been given by intravenous injection (IV). In 2 cases (case 1, 5), toluidine blue (TB) has been administered: In case 1 orally (PO) and IV, in case 5 only IV. In case 4, where a blue cleaning agent had been ingested, no detailed medical records were provided. Among the remaining non-hospital cases, in 2 cases the intake of Rohypnol^®^ was confirmed (cases 7, 8). For a compilation of all autopsy cases see Table 1.

**Table 1:** Overview of the cases

Case	Age / Sex	Weight [kg]	Substance	Dose (IV)	Time from administration to death	Coloured organs	Annotation
1	72 M	136	TB (PO and IV)	240 mg	2,5 h (IV)	serosa, brain, heart, stomach (mucosa), duodenum (mucosa)	oral and parenteral administration of TB at colonoscopy (PO) and during ICU treatment (IV)
2	30 M	119	MB (IV)	25 mg	2,5 h	brain, heart, lung (serosa)	parenteral administration of MB during ICU treatment
3	71 M	74	MB (IV)	50 mg / 50 mg / 100 mg	7 h / 6 h / 5 h	brain, heart, pancreas (serosa)	parenteral administration of MB during ICU treatment
4	79 F	73	Detergent (PO)	NR	25 h	urinary bladder (mucosa)	oral ingestion of C. I. Acid Blue 9-colored detergent
5	73 F	86	TB (PO)	NR	2 h	colon (mucosa)	oral administration at colonoscopy
6	40 M	154	MB (IV)	100 mg / 100 mg	10 h / 6 h	brain	parenteral administration of MB during ICU treatment
7	46 F	65	Rohypnol^®^ (PO)	NR	NR	stomach (mucosa)	oral ingestion of C. I. Acid Blue 74-colored drug
8	30 F	55	Rohypnol^®^ (PO)	NR	NR	stomach (mucosa), duodenum (mucosa)	oral ingestion of C. I. Acid Blue 74-colored drug
9	71 F	107	MB (IV)	100 mg / 100 mg	4,5 h / 1 h	brain, heart	parenteral administration of MB during ICU treatment
10	79 M	133	MB (IV)	NR	< 24 h	brain, heart	parenteral administration of MB during ICU treatment
11	75 M	89	MB (IV)	NR	< 24 h	urine	parenteral administration of MB during ICU treatment

NR: not reported; TB: Toluidinblue; MB: Methylenblue; IV: intravenous administration; PO: oral administration

### Review of the literature

Besides the known decay-typical greenish color changes, only some case reports and case series about blue-green discolorations of organs during autopsy have been published [2–6]. These works describe an initial subtle blue or blue-green coloration of organ surfaces and cut surfaces, which darkened within a short period of time. In these cases, administration of MB during medical therapy was seen as a possible cause. A subsequent search on publications investigating the administration of TB prior to death as possible cause for such discolorations did not yield any results. Greenish or bluish discolorations have also been described in cases of intoxication with hydrogen sulfide [7], after poisoning with dextropropoxyphene [8], ingestion of colored windscreen washer liquid [9] or Rohypnol^®^ [10].

## Discussion

The presented study was motivated by an autopsy case with initially unclear greenish to bluish organ discolorations. As is not uncommon in forensic autopsies, only little information on the medical history was initially provided. A subsequent retrospective case evaluation including a review of the medical records, and the literature revealed, that when it comes to green-blue discoloration of tissues or organs during autopsy, especially MB needs to be considered as a drug that could have been administered during medical therapy prior to death. Furthermore, TB and other colored drugs might cause such discolorations as well.

### Methylene blue (MB)

MB, also known as C.I. Basic Blue 9, was originally developed as a dye for the textile industry and is one of the first fully synthetic drugs to be used in medicine. Paul Ehrlich discovered the possibility of staining histological specimens with it and Robert Koch was the first to succeed in the microscopic visualization of tuberculosis bacteria with the help of MB staining [11]. Ehrlich and Guttmann first applied MB successfully as a drug for the treatment of malaria in 1891 [12]. Even though MB was later replaced by other drugs for this purpose [13], it was still used as an antimalarial drug by the US armed forces in the South Pacific until the end of World War II. However, MB was not very popular among the soldiers as it led to a blue coloration of the urine. In the memoirs of General Douglas MacArthur, commander of the Allied forces in the Southwest Pacific, it says: “Even at the loo we see, we pee, navy blue” [14]. After its use as an antimalarial drug, other medical indications followed. At the beginning of the 20th century, for example, MB was added to the medication of psychiatric patients to monitor their compliance based on the blue coloration of the urine. As an antidote, MB was initially used in the 1920s and 1930s for the treatment of cyanide poisoning [15] and later on for induced methemoglobinemia [16]. Nowadays, MB is still one of the most effective antidotes for the intravenous treatment of acquired methemoglobinemia [17, 18]. Probably the most common use of MB is as a dye in clinical examinations of the gastrointestinal and the urinary tract [19]. In surgery, MB is also used to visualize fistulous tracts, hollow organs and tissues (ureters, tumors) [20, 21].

Over the last two decades, studies in intensive care medicine have shown that MB can improve the hemodynamic situation in patients with therapy-refractory distributive shock [19, 22, 23] by reducing peripheral vasodilatation [24] and the mismatch between macro- and microcirculation [19].

Although the use of MB can improve hemodynamics in certain types of shock, no mortality benefit has yet been demonstrated in meta-analyses [25]. Surveys from the USA show that around 40% of (pediatric) intensive care physicians have already used or considered using MB in refractory shock [26]. To our best knowledge, up to now, no user data for adult intensive care medicine and no recommendations for the routine use of MB in acute shock exist in Germany. Therefore, systemic administration of MB in patients with refractory shock is presumably only used in individual cases. For the evaluated autopsy cases in which MB was administered intravenously (case 2, 3, 6, 9, 10, 11), it can be assumed that the patients received MB as ultima ratio treatment in states of shock.

During autopsies after antemortem administration of MB, green, greenish, green-blue, and turquoise discolorations of organs have been described in the literature, including the brain and the heart [2, 3], the spongiosa of the vertebrae [4], the parietal pleura [5] and the gastric mucosa [6]. Clinical observations of conspicuous color changes usually refer to a typical blue discoloration of the urine, which can also affect the stool [12]. In addition, some authors report temporary blue discoloration of the lacrimal fluid, sclera, saliva, mucous membranes and skin [14, 19, 27–29].

The observation at autopsy, where an initially subtle bluish discoloration of organs began to darken after a few seconds and turned into a strong green-blue discoloration is due to an oxidation process. In the presence of oxygen, the colorless leuco-MB turns into blue MB [5, 30]. In chemistry class, this reaction is known as the “blue bottle experiment” [31]. Depending on the way of application, MB can lead to discoloration of the mucosa in the gastrointestinal tract or to discoloration of entire organs. In the evaluated autopsy cases after clinically documented administration of MB, discoloration of the brain and heart were predominantly observed. In addition, the serosa of the lungs and pancreas were affected in one case each (Table 1). In conventional histological examinations, the autoptically visible staining by methylene blue is usually no longer detectable [5]. In the literature, only an implied faint visibility is described when using a special fixation technique [32]. Due to the retrospective nature of the study, the potential presence of MB in the tissues could not be proven by histological or toxicological examinations [4], which obviously is a limitation of the study. However, considering the literature and the clinical context, the authors have no reasonable doubt about the administration of MB as the cause of the discolorations. Beyond that, the study provides an indication of the visual detectability period of MB at autopsy. The reviewed cases included single and multiple administrations of MB, with the last administration having taken place at least 1 h and at most 5 h before death. A reliable visual post-mortem detectability period cannot be derived from the small number of cases, but case 11 may serve as an indication for the upper limit: The time of administration could not be narrowed down more exactly than less than 24 h before death and there was no more organ staining, but still a blue coloration of the urine.

### Toluidine blue (TB)

TB, also known as tolonium chloride or C.I. Basic blue 17, is an acidophilic metachromatic blue dye that has been known for various medical applications since its discovery by William Henry Perkin in 1856. However, TB is mainly found in the dye industry [33]. In medicine, TB is often used for in-vivo and in-vitro staining and visualization of tissue structures. It selectively stains acidic tissue components and has a high affinity for nucleic acids and thus accumulates in cell nuclei of tissues with a high DNA and RNA content [34]. The fact that dysplastic and neoplastic cells can contain quantitatively more nucleic acids than cells of healthy tissue led to the use of TB for staining mucosal epithelia in vivo as early as the 1960s [33]. In Germany, TB is approved for vital staining in chromoendoscopy, chromolaparoscopy, for intraoperative vital staining of epithelial corpuscles for the visualization of fistula ducts and as an antidote for severe methemoglobinemia [35]. In the forensic context, TB can be used in gynecological examinations to detect injuries [36].

In case 1 and case 5, TB has been given PO during colonoscopy. Additionally, in case 1, TB was administered IV. There is no data available that would support systemic administration of TB in a state of shock. While medical MB was temporarily unavailable in Germany due to supply shortages, TB has been used as an alternative antidote to MB [37]. This alternative treatment might have been the reason for IV administration in case 1, where TB has presumably been used as a substitute for MB in a state of shock.

The evaluated autopsy cases and the clinical literature [33] suggest that TB, like MB, can also lead to similar discolorations of organs at autopsy. Local mucosal staining, e.g. during endoscopic procedures, is clinically intended [35] and well known. After systemic administration of TB however, to our best knowledge, organ discolorations at autopsy have not been reported yet. The reason for this may be the rare systemic use of TB, as it has a more limited clinical spectrum of therapeutic indications than MB.

### Other drugs

In the three remaining cases without antemortem administration of MB or TB, the cause of death was intoxication with colored drugs or cleaning products: In two cases (case 7, 8) a mixed intoxication with Rohypnol^®^ and in one case (case 4) an intoxication with a formalin-containing cleaning agent with blue warning color.

Rohypnol^®^ tablets contain the intermediate-acting benzodiazepine flunitrazepam. In the past, Rohypnol^®^ has been used illegally as an aid to sexual assault (known as a “date rape drug”) and was unknowingly given to victims in their drinks. Therefore, the manufacturer added the blue dye indigocarmine (C.I. Acid Blue 74, E 132) to the tablets which turns clear liquids into blue in order to warn potential victims. The ability of this dye to also stain the gastric mucosa blue has already been described in the literature [10]. In cases 7 and 8, it must be assumed that the discoloration of the gastric wall and the doudenal mucosa observed on autopsy is due to the blue dye indigocarmine.

In case 4, the blue warning color (dye: Acid Blue 9, E 133, Brilliant Blue FCF) in the detergent consumed obviously led to an isolated discoloration of the urinary bladder mucosa. The gastrointestinal tract was not or no longer affected, and no other organs showed any suspicious discoloration. As described in a case report, the ingestion of this dye, which is also used as a food additive, can lead to a green discoloration of the urine [35]. Therefore, it can be assumed that the blue dye of the detergent led to an isolated discoloration of the urinary bladder.

## Conclusion

If a green-blue discoloration of organs is noticed during the autopsy, an administration of certain xenobiotics is to be considered. The affected organs provide an initial indication of the possible route of application and the type of substance. If only the upper gastrointestinal tract is affected, an oral intoxication with toxins or drugs with warning or signal color should be considered. If there is discoloration of organs, especially the brain and heart, a medical intravenous administration of toluidine blue or methylene blue is likely. A differentiated interpretation of the etiology of such conspicuous discolorations at autopsy should be made considering the (medical) history of the corpse and, if necessary, supplementary (toxicological) examinations.

## Key points

1. Depending on the route of administration, xenobiotics taken during life can cause unusual discoloration of various organ systems at autopsy and provide clues to medical treatment or intoxication.
2. After systemic administration of methylene blue and toluidine blue in the intensive care unit, a darkening blue-green discoloration was observed in autopsies, particularly of the brain and heart.
3. Orally given or ingested drugs and other foreign substances containing warning colors such as indigo carmine (E132) or brilliant blue FCF (E133) led to discoloration of the upper gastrointestinal tract or urinary bladder.

## Data Availability

Data for this study can be requested by contacting the corresponding author.
